# Liquid-liquid phase separation and its regulation of the BACH2 intrinsically disordered region

**DOI:** 10.2142/biophysico.bppb-v23.0010

**Published:** 2026-02-25

**Authors:** Hironori Hayashi, Miyuki Kato-Murayama, Takeshi Kurasawa, Eiichi N. Kodama, Mikako Shirouzu, Kazuhiko Igarashi, Kazutaka Murayama

**Affiliations:** 1 Division of Infectious Diseases, International Research Institute of Disaster Science, Tohoku University, Sendai, Miyagi 980-8572, Japan; 2 Laboratory for Protein Functional and Structural Biology, RIKEN Center for Integrative Medical Sciences, Yokohama, Kanagawa 230-0045, Japan; 3 Department of Biochemistry, Tohoku University Graduate School of Medicine, Sendai, Miyagi 980-8575, Japan; 4 Department of Infectious Diseases, Tohoku University Graduate School of Medicine, Sendai, Miyagi 980-8575, Japan; 5 Tohoku Medical Megabank Organization, Tohoku University, Sendai, Miyagi 980-8573, Japan; 6 Division of Biomedical Measurements and Diagnostics, Graduate School of Biomedical Engineering, Tohoku University, Sendai, Miyagi 980-8575, Japan

**Keywords:** intrinsically disordered protein, condensation, heme binding, phosphorylation, TBK1

## Abstract

Intrinsically disordered regions (IDRs) are vital for several cellular processes. They play a significant role in liquid-liquid phase separation (LLPS). LLPS enhances reaction efficiency by locally concentrating proteins and nucleic acids. The BACH2 transcription factor contains such IDRs. Early observations hinted at the phase separation capabilities of BACH2. However, how BACH2-IDR directly participates in LLPS is not fully understood. Its precise modulation by heme binding and phosphorylation also requires further elucidation. This study experimentally demonstrated the intrinsic capability of BACH2-IDR (residues 331–520) to undergo LLPS *in vitro*. Microscopic observations revealed that TANK-binding kinase 1(TBK1)-mediated phosphorylation suppressed LLPS of BACH2-IDR. Conversely, the coexistence of heme enhanced LLPS, initiating LLPS at lower polyethylene glycol concentrations. Bioinformatic analyses supported these experimental observations. Tools such as FuzDrop and CIDER were used to predict LLPS propensity from sequences. Specifically, the calculations for the sequence of phosphorylation-mimic mutations showed changes in LLPS-promoting regions. Heme binding influenced TBK1-mediated phosphorylation sites. Some of these sites overlap with predicted LLPS-driving regions. Heme binding also induced substantial conformational alterations within BACH2-IDR. Consequently, we propose LLPS as a fundamental regulatory mechanism for BACH2 protein function. This process complements its known regulation through heme binding and phosphorylation.

## Significance

This study demonstrated that the BACH2 intrinsically disordered region undergoes liquid-liquid phase separation (LLPS), a novel regulatory mechanism for this critical transcription factor. We show that heme binding enhanced LLPS, whereas TBK1-mediated phosphorylation suppressed it, revealing how physiological signals fine-tune BACH2 activity. We propose LLPS as a fundamental regulatory mechanism alongside heme binding and phosphorylation. Our findings provide crucial insights into the functional control of BACH2, explaining past observations of dynamic nuclear foci and highlighting a new layer of protein regulation.

## Introduction

Intrinsically disordered proteins (IDPs) and their intrinsically disordered regions (IDRs) are prevalent throughout eukaryotic proteomes, fulfilling indispensable roles in a multitude of cellular processes [1,2]. IDPs/IDRs exist as dynamic ensembles of interconverting structures [3]. This intrinsic flexibility, coupled with their unique amino acid composition, enables them to perform diverse functions including molecular recognition, signaling, and gene regulation [1]. A particularly significant and rapidly evolving research area of IDP/IDR function is their involvement in liquid-liquid phase separation (LLPS) [3]. LLPS is a fundamental biophysical process that enhances the efficiency of various reactions by locally concentrating specific proteins and nucleic acids [2]. Their assembly and disassembly are precisely regulated, often in response to environmental cues, underscoring their transient and functionally adaptive nature [2]. Phosphorylation of IDRs is a well-established mechanism for fine-tuning protein function, often by inducing conformational changes that influence binding affinity, protein stability, and cellular localization [4]. For instance, phosphorylation can significantly alter the propensity of IDRs to undergo phase separation or aggregation, effectively modifying their liquid-liquid phase behavior [4].

BTB and CNC homolog 2 (BACH2) and its paralogue BACH1 define a transcription factor family of the BTB-basic region leucine zipper (bZip) [5–7]. The bZip domain is central to DNA binding and frequently forms heterodimers with small Maf proteins [7,8], whereas the BTB domain mediates protein-protein interactions [9]. However, notable amounts of BACH2 and BACH1 are spanned by IDRs. The regulatory activities of BACH2 are closely linked to cellular responses to oxidative stress and acquired immune responses of B and T lymphoid cells [5,10]. Its target genes include *Prdm1*, the key regulatory gene for plasma cell and effector T cell differentiation, and *Hmox1* encoding heme oxygenase-1 (HO-1). A distinctive regulatory mechanism governing BACH2 and BACH1 involves a direct interaction with heme. Both proteins contain multiple Cys-Pro (CP) motifs nested within their intrinsically disordered regions that act as primary heme-binding sites [11,12]. Heme can bind to these motifs through various coordination modes, including a specific 5-coordinated mode characteristic of CP motifs and a more general 6-coordinated mode [13]. This binding event induced significant conformational alterations in BACH2-IDR (aa331–520, hereafter referred to as BACH2-IDR^331–520^). In addition to heme binding, post-translational modifications, particularly phosphorylation, represent another vital step in BACH2 regulation. TANK-binding kinase 1 (TBK1) has been identified as a kinase that both interacts with and phosphorylates the IDR of BACH2 [14]. This phosphorylation is pivotal for inactivating BACH2 and modulating its interactions with other proteins, including its corepressor NCOR1 and the ubiquitin E3 ligase adaptor, FBXO22. TBK1 phosphorylates multiple serine and threonine residues of BACH2, some of which are heme-regulated (HR-5), whereas others are non-heme-regulated (NHR-5). Therefore, heme and phosphorylation appear to interact to regulate the IDR of BACH2 [14].

Observations of BACH2 behavior have provided early hints of its phase separation capabilities, leading to a widespread understanding of LLPS in cell biology. Our previous work in 2004 reported the formation of dynamic BACH2 “nuclear foci” in response to oxidative stress [15]. Importantly, these foci were characterized by their rapid turnover and direct involvement in repressing transcriptional activity associated with PML nuclear bodies. The observed dynamic nature and functional role of these foci align strikingly with the defining characteristics of biomolecular condensates formed through LLPS [2]. A seminal study that established LLPS as a key mechanism for membraneless organelle formation was published several years later in 2009 [16]. Despite these early findings and the wealth of recent knowledge concerning IDRs and LLPS, a comprehensive understanding of how the IDR of BACH2 directly participates in LLPS and how this process is precisely modulated by crucial physiological signals, such as heme binding and phosphorylation, remains to be fully elucidated. This study aimed to experimentally demonstrate the intrinsic capability of BACH2-IDR^331–520^ to undergo LLPS. We investigated the impact of heme binding and TBK1-mediated phosphorylation on the phase separation behavior of BACH2-IDR^331–520^.

## Materials and methods

### Observation of BACH2 condensation in Hela cells

The mice BACH2–EGFP construct was cloned into the pLV vector under control of the CMV promoter and enhancer. HeLa cells were cultured in 35-mm dishes and transfected with FuGENE® HD (Promega) according to the manufacturer’s instructions. The cells were fixed with 4% paraformaldehyde 24 hours after transfection and imaged using a Zeiss LSM 780 confocal microscope equipped with a Plan-Apochromat 63×/1.4 oil-immersion objective.

### Protein expression and purification

Sequences encoding the intrinsically disordered region of BACH2 (residues 331–520) with a GFP fusion protein at the C-terminus (BACH2-IDR^331–520^-GFP) were cloned into the pCR2.1-TOPO vector (Thermo Fisher Scientific). The histidine tag was attached at the N-terminus of BACH2, and BACH2 and GFP were connected through the linker sequence “SGPSSGLEVLFQGP,” including PreScission protease (Cytiva) recognition site. BACH2-IDR^331–520^-GFP was expressed in a cell-free expression system [17,18]. The protein was purified using a HisTrap column (Cytiva, Tokyo, Japan) with 20 mM Tris-HCl buffer (pH 8.0) containing 800 mM NaCl and 20 mM imidazole and eluted with 500 mM imidazole, and subjected to TEV protease digestion (4°C, overnight) to cleave the N-terminal histidine-tag. Proteins were purified using HiTrap Q and Superdex 75 gel filtration chromatography (Cytiva). The purified protein was concentrated in 20 mM Tris-HCl buffer (pH 8.0), 150 mM NaCl, and 1 mM dithiothreitol (DTT) to a final concentration of 3~3.3 mg/mL by Amicon Ultra4 (Millipore Sigma). The purified samples were evaluated using MALDI-tof-MS (AXIMA Performance, Shimadzu) with a single peak: MW 49248 (theoretical 49197).

A BACH2 sample was prepared without GFP (BACH2-IDR^331–520^) to exclude the possible effects of GFP on LLPS. The GFP domain of BACH2-IDR^331–520^-GFP was cleaved using PreScission protease in 20 mM Tris-HCl buffer (pH 8.0), 150 mM NaCl, 2 mM DTT, and 20 mM imidazole at 4°C overnight. BACH2-IDR^331–520^ was purified as previously described (HisTrap and TEV digestion). The purified protein was concentrated in 20 mM Tris-HCl buffer (pH 8.0), 150 mM NaCl, and 2 mM TCEP, to a final concentration of 4.7 mg/mL.

TBK1 was cloned into the expression vector pFastBac1 (Thermo Fisher Scientific) and expressed in Sf9 cells using the Bac-to-Bac Baculovirus Expression System (Thermo Fisher Scientific). The protein was purified using chromatography with a HisTrap column (Cytiva) with 20 mM Tris-HCl buffer (pH 8.0) containing 800 mM NaCl and 20 mM imidazole, and eluted with 500 mM imidazole. After buffer exchange to 20 mM Tris-HCl buffer (pH 8.0) and 1 mM DTT, the sample was applied to anti-FLAG M2 agarose (MilliporeSigma) mixed at 4°C overnight. Next, the sample was washed with 20 mM Tris-HCl buffer (pH 8.0), 150 mM NaCl, and 1 mM DTT and eluted with 150 μg/mL 3× FLAG peptide. The eluted sample was concentrated to 1.4 mg/mL using Amicon Ultra-4. The final sample was added to 10% glycerol and stored at –80°C before use.

### BACH2-IDR^331–520^ phosphorylation by TBK1

The phosphorylation of BACH2-IDR^331–520^ by TBK1 was performed in a mixture containing 75 μL of 15 μM BACH2-IDR^331–520^, 38 μL of 0.5 μM TBK1, and 967 μL of the kinase buffer (Cell Signaling Technology, supplemented with 0.1 mM ATP and 0.5 mM TCEP). The reaction was conducted at 30°C for 30 min, and terminated by adding 25 mM EDTA.

### Observation of liquid-liquid phase separation

Initially, LLPS was observed in BACH2-IDR^331–520^-GFP at final concentrations of 2.5, 5, 7.5, and 10% PEG8000. Fluorescence images were obtained using EVOS FL Auto (Thermo Fisher Scientific) with excitation and emission wavelengths of 470 and 525 nm, respectively. The solutions to observe LLPS for BACH2-IDR^331–520^ were prepared by mixing protein solution (2.5 mg/mL final concentration), 50% PEG8000, and heme solution in DMSO (with heme, 20 μM final) or DMSO (without heme). Solutions were prepared at final concentrations of 2, 4, 6, 8, 10, and 12% PEG8000. The LLPS solution was finally adjusted to 20 μL with buffer solution (same as the protein solution) and stored at room temperature (25°C) for 90 min. Images of LLPS were captured using EVOS FL Auto microscope in transmitted light mode. The number of droplets was evaluated using ImageJ (Rasband, W.S., ImageJ, U. S. National Institutes of Health, Bethesda, Maryland, USA, https://imagej.net/ij/, 1997–2018) with images covering the entire field of view. Bright-field images were converted to 8-bit, contrast-enhanced, and circular objects were quantified using particle analysis in ImageJ.

### Calculations of physicochemical properties from sequences

To investigate the physicochemical properties of the IDR of BACH2, sequence analyses were performed using software with standard protocols: CIDER [19], FuzDrop [20–22], and Albatross [23]. The BACH2-IDR sequences for analyses were prepared in the region of 331–520 for the native sequence, as well as the phosphorylation-mimic sequences in which phosphorylation sites were mutated to aspartic acid. These mutation sites were non-heme-regulated (NHR-5): S342, T347, S352, S464, and S472, and heme-regulated (HR-5): S352, S357, T382, S402, and S472 [14].

## Results and discussion

### Liquid-liquid phase separations induced by BACH2 within cells

**Figure 1:**
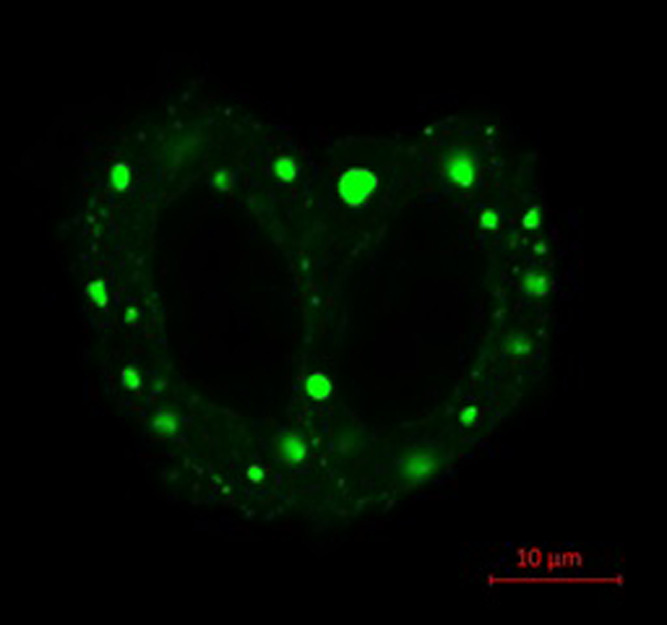
Observation of “condensations” of BACH2 in cells.

As mentioned above, our previous study in 2004 suggested condensations of BACH2 in cells. The first report as “liquid-liquid phase separation” was in 2009, and we did not mention nuclear foci as a LLPS at that time. In the present study, we confirmed that LLPS was induced by BACH2. The C-terminal EGFP-labeled BACH2 was overexpressed in HeLa cells. As this experiment was performed without any stimuli that would induce the nuclear accumulation of BACH2, BACH2-EGFP was observed as droplets in the cytoplasm (Figure. 1).

### Liquid-liquid phase separations were observed in BACH2-IDR^331–520^

**Figure 2:**
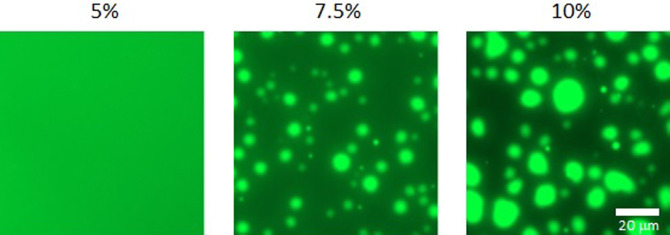
Fluorescence measurements of liquid-liquid phase separations of GFP fusion protein of BACH2 intrinsically disordered region (331–520). Liquid-liquid phase separations were observed as green fluorescence at different polyethylene glycol concentrations (5, 7.5, and 10%).

**Figure 3:**
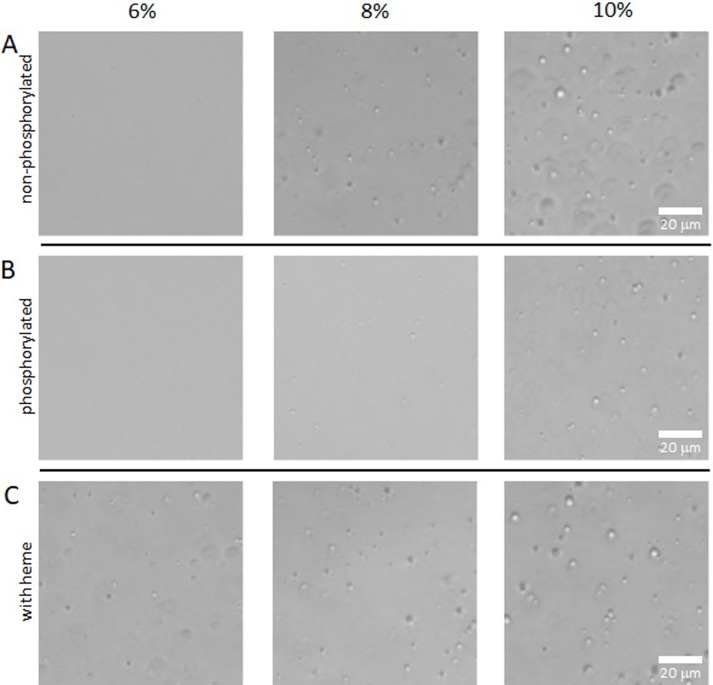
Liquid-liquid phase separations of BACH2 intrinsically disordered region (331–520). Phase separations were observed for the non-phosphorylated (A), phosphorylated (B), and non-phosphorylated samples with heme (C).

In our previous studies of BACH proteins, we paid close attention to the intrinsically disordered state of the protein. Because larger protein fragments containing the IDRs of BACH2 were difficult to purify, we focused on a subfragment of IDR spanning residues 331–520. The BACH2-IDR^331–520^ and C-terminal GFP fusion proteins were prepared for observation using fluorescence microscopy. Under our experimental conditions, the LLPS of BACH2-IDR^331–520^-GFP was observed in 7.5% PEG8000 (Figure. 2). Although fluorescence observations confirmed that the GFP fusion protein BACH2-IDR^331–520^-GFP undergoes LLPS depending on the concentration of polyethylene glycol, the extent to which LLPS is affected by GFP is unknown. We then prepared BACH2 proteins without GFP for observation, focusing on BACH2-IDR^331–520^. GFP-truncated samples were purified and concentrated using BACH2-IDR^331–520^. Phase separation was also observed for BACH2-IDR^331–520^ depending on the polyethylene glycol concentration (Figure. 3a). The phase separation was visually observed in 8% PEG8000. These results confirm that LLPS is caused by BACH2-IDR^331–520^

LLPS experiments were also performed on phosphorylated (Figure. 3b) and heme-coexisting samples (Figure. 3c). BACH2-IDR^331–520^ was incubated with TBK1 and ATP for its phosphorylation. Both samples exhibited LLPS depending on PEG8000 concentration. However, when comparing the images at each concentration, LLPS appeared differently. In the phosphorylated sample, LLPS appeared in 8% PEG8000, but seemingly contained smaller droplets compared to the non-phosphorylated sample. In contrast, samples containing heme showed LLPS from 6% PEG8000. To quantitatively evaluate the initial formation of these droplets, we counted the droplet numbers using ImageJ. At a 6% PEG concentration, the droplet counts were 39 (non-phosphorylated), 0 (phosphorylated), and 91 (heme-coexisting), respectively. At an 8% PEG concentration, the counts were 117, 84, and 95. These results indicate that the propensities for LLPS initiation are heme-containing>non-phosphorylated>phosphorylated. Although it was difficult to make a quantitative argument for LLPS as a function of the concentration of PEG8000 in this experiment, both phosphorylation and heme qualitatively altered the concentration dependence of PEG8000 at which droplets were produced. In other words, the LLPS of BACH2-IDR^331–520^ was suppressed by phosphorylation and enhanced by heme-coexistence compared with “intact” BACH2-IDR^331–520^.

### Sequence analyses support phase separation propensity for BACH2-IDR^331–520^

**Figure 4:**
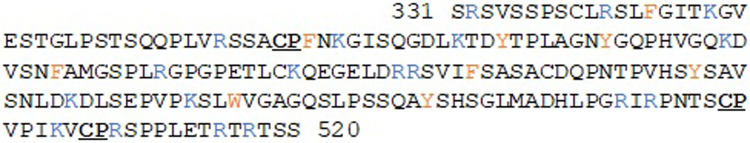
Sequence of BACH2 intrinsically disordered region used in this study. Letters in blue and orange indicate basic amino acids (Lys and Arg) and amino acids with aromatic residues (Phe, Tyr and Trp), respectively. The Cys-Pro motifs are underlined.

In our previous studies, we revealed that BACH2-IDR^331–520^ possesses unique amino acid content [24], including high contents of serine and proline. These amino acids play critical roles in promoting LLPS by providing flexibility and interaction sites for proteins and multivalent interactions are important [3,25]. Aromatic and basic amino acids are key factors in the formation of cation-π interactions in LLPS [26], and BACH2-IDR^331–520^ includes four tyrosine, four phenylalanine, one tryptophan, eight lysine, and eleven arginine residues (Figure. 4). Based on the “sticker and spacer” model [27], these residues should be dispersed throughout the sequence. Although we could not quantitatively show the degree of amino acid dispersion, aromatic and basic amino acids appeared to be qualitatively well dispersed as stickers. In addition, the high abundance of glycine and serine, which are spacers, suggests that the cation-π interaction consisting of aromatic and basic amino acids is expected to be an effective interaction for the LLPS of BACH2-IDR^331–520^.

**Figure 5:**
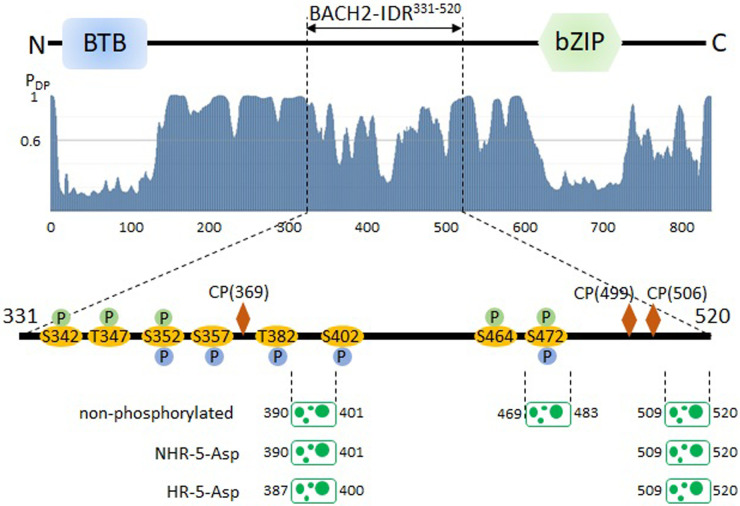
Phosphorylation and “droplet-driver” regions in BACH2. Top: the two structural domains in BACH2 are indicated as “BTB” and “bZIP.” Middle: the phase separation probability for whole region of BACH2 is depicted as the blue histogram of the probability value between 1 (high probability) to 0 (low probability). Bottom: the phosphorylation sites and droplet-driver regions are indicated on the BACH2 intrinsically disordered region. Two types of phosphorylated sites are labelled “P” with green circles (non-heme regulated site, NHR-5) and with blue circles (heme regulated site, HR-5). LLPS-promoting regions indicated using FuzDrop are depicted by green illustrations with sequence positions (the numbers on the left and right). Positions of Cys-Pro motifs are indicated by the brown rhombi.

Using bioinformatic analysis, we predicted the propensity for LLPS using sequence data. In this study, we used FuzDrop [21] and CIDER [19] to predict the propensity of BACH2-IDR^331–520^ to develop into LLPS. FuzDrop suggested that the region 331–520 has the propensity of phase separation (Figure. 5) with its probability value (p_LLPS_: the probability of forming a droplet state through LLPS) 0.6416, indicating a “droplet-driver” with p_LLPS_>0.6 as defined in the FuzDrop program. Although the sequence region 331–520 includes droplet-promoting regions (390–401, 469–483, and 509–520), according to the plot of the probability of droplet-promoting residues, the probability is rather low compared to that of the whole sequence (Figure. 5). Focusing on the entire sequence (1–839), a more suitable region for LLPS was observed adjacent to the N-terminal side (140–330). The probability of LLPS in this region was p_LLPS_=0.9972, which was a high score.

### Phosphorylation and heme binding are involved in the phase-separation of BACH2-IDR^331–520^

Phosphorylation in the above experiment reflects phosphorylation without heme; therefore, S342, T347, S352, S464, and S472 (NHR-5) are the phosphorylation sites that are prone to occur under the experimental conditions. Microscopic observation revealed that phosphorylated BACH2 caused LLPS itself but was less promotive than those without phosphorylation. To assess the effects of phosphorylation using bioinformatics tools, two phosphorylation-mimic sequences in which the phosphorylation sites were substituted with aspartic acid, NHR-5-Asp, and HR-5-Asp, were prepared to introduce negative charges into FuzDrop and CIDER calculations (Figure. 5). FuzDrop analysis indicated that the LLPS-promoting region was altered. Compared to non-phosphorylated BACH2-IDR^331–520^, NHR-5-Asp and HR-5-Asp lacked region 469–483 (Figure. 5), and the LLPS probabilities decreased to p_LLPS_=0.5366 and 0.6021, respectively. CIDER can calculate some physicochemical parameters, and we focused on the factor for charge distribution κ, which is correlated with the propensity for LLPS [19]. The larger the value, the greater the tendency for phase separation. The value for each sequence was as follows: 0.134 for non-phosphorylated, 0.126 for NHR-5-Asp, and 0.149, HR-5-Asp. These results are consistent with our observations for the phosphorylated sample, which can be regarded as NHR-5-Asp. Although we could not experimentally investigate LLPS in HR-5, the LLPS propensity be expected to HR-5>NHR-5.

We previously investigated heme binding to the BACH proteins (BACH1 and BACH2) [13,28–31]. Heme binding to the IDR of BACH2 may induce conformational changes, which in turn alter protein-protein interactions, such as those with TBK1 [14]. Heme binding alters TBK1-mediated phosphorylation sites in BACH2-IDR^331–520^. Compared with conditions without heme, in the presence of heme, the phosphorylation sites were altered to serine/threonine residues around the N-terminal CP motif (Cys369-Pro370) (Figure. 5). The results for BACH1 showed that its Cys438-Pro439 motif primarily bound to heme [14], and this CP motif may correspond to the CP motif (Cys369-Pro370) in BACH2. These sites were adjacent to the ‘droplet-driver’ region (390–401) (Figure. 5), strongly suggesting that heme-binding-phosphorylation influences LLPS. Moreover, in BACH1, 5-coordinated heme binding, which is specific to the CP motif, correlates with 6-coordinated heme binding, enabling the presence of 5-coordinated heme binding to increase to 6-coordinated heme binding [28]. Because 6-coordinated heme binding can also occur non-specifically, intermolecular 6-coordinated bonds may bring molecules closer together and increase intermolecular interactions, thereby promoting LLPS compared to conditions without heme.

### BACH2-IDR^331–520^ is the regulatory region through phase separation

The global physicochemical parameters of BACH2-IDR^331–520^ in solution were predicted using various software tools. CIDER predicts protein conformations and plots them on a conformational diagram based on sequence features. Calculations for three BACH2-IDR^331–520^ sequences, non-phosphorylated, phosphorylation mimic NHR-5-Asp and HR-5-Asp, classified all of them as “Globules & Tadpoles,” indicating a degree of compactness. ALBATROSS, which can estimate the radius of gyration (Rg) directly from the sequence, calculated Rg in the range 43.9–43.7 Å for these three sequences. For non-phosphorylated BACH2-IDR^331–520^, Small-Angle X-ray Scattering (SAXS) measurements resulted in an Rg of 40.1 Å [13], indicating a more compact structure for a complete IDR. Conventionally, protein compactness is considered unfavorable for driving LLPS [23]. FuzDrop for the 331–520 region of BACH2 showed a relatively low propensity for p_LLPS_ (Figure. 5). FuzDrop considers p_LLPS_>0.6 as a benchmark for a “droplet-driver,” suggesting that the BACH2-IDR^331–520^ results indicate a moderate propensity as a droplet-driver. The moderate tendency for a droplet-driver likely suggests the possibility of selective interaction with other proteins (not only self-association), depending on the circumstances. In the context of the functional regulation of proteins, the modulation of protein-protein interactions, such as self-association and recruitment of other proteins, is considered a significant advantage. BACH2-IDR^331–520^ regulates its function through its intrinsic conformational properties as an intrinsically disordered protein (displaying a degree of compactness), heme binding, and phosphorylation [14]. Therefore, LLPS is considered the third functional regulatory mechanism of BACH2.

## Conclusion

We demonstrated that BACH2-IDR^331–520^, which is responsible for heme binding and phosphorylation, underwent LLPS by observing the phase separation of isolated BACH2-IDR^331–520^ *in vitro*. Phosphorylation was seemingly less effective for the LLPS of BACH2-IDR^331–520^, whereas heme coexistence promoted it, initiating LLPS at lower polyethylene glycol concentrations. Sequence analyses supported this intrinsic propensity, predicting that “droplet-driver” regions within BACH2-IDR^331–520^ possess characteristics favorable for LLPS. In addition, phosphorylation-mimicking mutations in some residues of NHR-5 or HR-5 resulted in a reduced propensity for LLPS and altered LLPS-promoting regions. These findings suggest that both phosphorylation and heme binding are regulatory mechanisms for the phase separation of BACH2-IDR^331–520^. Heme binding can alter the phosphorylation sites that overlap with LLPS-driving regions. Consequently, we propose that LLPS is the third mechanism that regulates BACH2 protein function, along with heme binding and phosphorylation.

## Conflict of interest

The authors declare that they have no conflicts of interest.

## Author contributions

K. M. and K. I. conceived and designed the study. H. H. and E. N. K. conducted microscopic analysis of images. M. K. M. and M. S. prepared the protein samples. K. M. and K. I. drafted the manuscript.

## Data availability

The data generated and/or analyzed in the current study are available from the corresponding author upon reasonable request.
